# Application of Artificial Intelligence to Evaluate the Fresh Properties of Self-Consolidating Concrete

**DOI:** 10.3390/ma14174885

**Published:** 2021-08-27

**Authors:** Yuping Feng, Masoud Mohammadi, Lifeng Wang, Maria Rashidi, Peyman Mehrabi

**Affiliations:** 1 School of Civil Engineering, Northeast Forestry University, Harbin 150040, China; 2 Centre for Infrastructure Engineering, Western Sydney University, Penrith, NSW 2751, Australia; m.mohammadi@westernsydney.edu.au (M.M.); m.rashidi@westernsydney.edu.au (M.R.); peyman804m@gmail.com (P.M.)

**Keywords:** artificial intelligence, metaheuristic algorithm, superplasticizer demand, self-consolidating concrete

## Abstract

This paper numerically investigates the required superplasticizer (SP) demand for self-consolidating concrete (SCC) as a valuable information source to obtain a durable SCC. In this regard, an adaptive neuro-fuzzy inference system (ANFIS) is integrated with three metaheuristic algorithms to evaluate a dataset from non-destructive tests. Hence, five different non-destructive testing methods, including J-ring test, V-funnel test, U-box test, 3 min slump value and 50 min slump (T50) value were performed. Then, three metaheuristic algorithms, namely particle swarm optimization (PSO), ant colony optimization (ACO) and differential evolution optimization (DEO), were considered to predict the SP demand of SCC mixtures. To compare the optimization algorithms, ANFIS parameters were kept constant (clusters = 10, train samples = 70% and test samples = 30%). The metaheuristic parameters were adjusted, and each algorithm was tuned to attain the best performance. In general, it was found that the ANFIS method is a good base to be combined with other optimization algorithms. The results indicated that hybrid algorithms (ANFIS-PSO, ANFIS-DEO and ANFIS-ACO) can be used as reliable prediction methods and considered as an alternative for experimental techniques. In order to perform a reliable analogy of the developed algorithms, three evaluation criteria were employed, including root mean square error (RMSE), Pearson correlation coefficient (r) and determination regression coefficient (R^2^). As a result, the ANFIS-PSO algorithm represented the most accurate prediction of SP demand with RMSE = 0.0633, r = 0.9387 and R^2^ = 0.9871 in the testing phase.

## 1. Introduction

Over the past few years, several studies were carried out to investigate the relationship between the percentage of mixing pozzolanic materials with cement and water for obtaining the optimum water-to-cement ratio in different types of concrete. Many researchers have worked on the application of cement replacements, such as fly ash, silica fume and slag, in concrete mixes [1,2,3,4,5]. The effect of natural powders, such as pumice, rice husk ash (RHA) and perlite on concrete properties, have been also investigated [6,7,8,9]. Incorporating concrete with pumice led to a higher strength-to-weight ratio in comparison with concrete with cement [10,11,12,13,14,15]. Slag is another most used cement replacement powder, which can provide some benefits such as low heat in hydration, proper performance, resistance to sulfate attack, acid, abrasion and corrosion [16].

Self-consolidating concrete (SCC) is one of the common types of concrete, which has been used in structural applications due to suitable workability and the spreading ability without the need for mechanical vibration [17]. Using a lower value of coarse aggregates in comparison to fine aggregates and higher cement content leads to better compactability and lower segregation and enhances the workability of concrete. Compactability can be enhanced by working on SCC mix design. In order to obtain a sustainable SCC, a reliable mix design is required. Slump retention is a critical parameter in SCC mix design, which has been widely investigated in recent years. One of the governing parameters in controlling slump retention is superplasticizer (SP) demand. Slump loss is the difference between measured slumps at various times of concrete production. The required amount of SP in SCC is directly related to the slump loss so that by increasing slump loss, more SP demand is required. Bani Ardalan et al. [18] studied the workability and compressive strength of SCC incorporating pumice, slag and fly ash as supplementary cementitious materials. The cement replacements proportions included 10% to 50% as binary mix designs. Silica fume was also used along with pumice in some mixes as ternary mix designs. It was found that the properties of mixtures were improved with silica fume incorporation. Shariati et al. [19] showed that using pumice powder and slag as cement replacements in SCC leads to promising results. However, more explorations on the amount of SP are still required to produce concrete with higher workability and placeability. There are limited non-destructive tests available for concrete, which are mostly related to fresh properties. Five key design parameters of SCC, including J-ring, U-box, V-funnel, 3 min slump and 50 min slump (T50), have a non-destructive nature.

Partial replacement of cement in SCC with other materials changes the fresh and mechanical properties. Although these changes can be observed by experiments, it is not simple to identify the most influential parameters on fresh properties and predict the design parameters. To address this problem, artificial intelligence (AI) models can be employed, which are able to produce accurate results by simulating human intelligence [20,21,22,23,24]. Uysal and Tanyildizi [25] used an artificial neural network (ANN) to predict the compressive strength of SCC with mineral additives. They found that the ANN model can be an appropriate alternative for experimental methods. Asteris et al. [26] applied ANN models to evaluate the mechanical properties of SCC based on the experimental data. It was reported that the back propagation neural networks are able to provide reliable results for predicting the compressive strength of SCC. Nguyen et al. [27] employed two AI algorithms to predict the compressive strength of fiber-reinforced high-strength SCC. The results indicated that the ANN model was more accurate compared to the ANFIS method. Golafshani et al. [28] combined ANN and ANFIS with GWO to develop the hybrid algorithms for predicting the compressive strength of normal strength concrete and high-performance concrete incorporating fly ash and blast furnace slag. It was indicated that hybrid models can increase the accuracy of the prediction. Douma et al. [29] developed an ANN model to predict the fresh properties and compressive strength of SCC incorporating fly ash. It was reported that the artificial neural network is an appropriate technique for evaluating the properties of SCC. Elemam et al. [30] studied the fresh properties and compressive strength of SCC containing limestone powder, silica fume and fly ash using an ANN model. It was concluded that the proposed optimization model is able to determine the optimum values of variables to achieve the desirable properties of SCC. Azimi-Pour et al. [31] deployed linear and non-linear support vector machine models to predict the compressive strength and fresh characteristics of high volume fly ash SCC. They deduced that compared to other kernel functions, the results of the SVM with radial basis function are more reliable.

Adaptive neuro-fuzzy inference system (ANFIS) as an intelligence method can learn and adapt automatically to solve optimization problems. In contrast to most analytical approaches, ANFIS does not require identifying the system parameters and therefore can find simpler solutions for multivariable problems. ANFIS is able to produce the best estimate circumstances by eliminating the vagueness in the process via excluding some input parameters. The ANFIS network is used to transform the compound performance characteristics into a single performance index. Generally, fuzzy systems are applied to interpret and assess the experimental data. However, some shortcomings in the accuracy and versatility of ANFIS have been identified, which were addressed by incorporating classic computing algorithms and optimization techniques [32,33,34,35,36]. Kennedy and Eberhart [37] introduced particle swarm optimization (PSO) in 1995, which was an intelligence evolutionary algorithm stimulated by the social behavior of bird flocking or fish schooling. This algorithm has a remarkable convergence rate compared to other evolutionary algorithms. Besides, ant colony optimization (ACO) is a continuous space algorithm inspired by observations on ant colonies, which show that they are social insects living in colonies where colony survival is more important than the survival of a component. One of the main aspects of ants’ behavior is their performance in finding food, particularly detecting the shortest path between food sources and nests. This mass intelligence aspect in ant behavior has attracted scientists’ attention to use ACO in different applications. On the other hand, differential evolution optimization (DEO) algorithm was presented to overwhelm the primary flaw of the genetic algorithm, namely the lack of local search. In the last few years, different types of AI models have been applied to predict and evaluate the performance of structural elements using experimental data [38,39,40,41,42].

Several AI-based models have been utilized to predict the hardened and fresh properties of concrete. Studies have revealed that using hybrid algorithms in many cases can lead to improving the performance of predictive models [43,44,45,46]. The main objective of this paper is to predict the SP demand of specific SCC by soft computing methods to avoid mathematical approaches with high nonlinearity. For this purpose, ANFIS algorithm is developed and combined with three metaheuristic algorithms, namely particle swarm optimization (PSO), ant colony optimization (ACO) and differential evolution optimization (DEO). Besides, the verified experimental data from the literature [18,19] are used to obtain an appropriate replacement for cement and optimize it by reducing environmental pollution and increasing the durability and properties of fresh concrete. In addition, the results of hybrid algorithms are compared and interpreted to determine the best one.

## 2. Experimental Methodology

A verified series of non-destructive test data has been derived from the literature [18,19] and employed in the soft computing process. In this section, for better understanding, some significant data and non-destructive test procedures are presented.

### 2.1. Materials

Pumice, fly ash, slag and silica fume were utilized as the cement alternatives at various replacement percentages and applied in binary and ternary mixtures. Table 1 summarizes the specific density and chemical components of the considered cement.

### 2.2. Mix Proportion

The first series of mix designs consists of fly ash, pumice and slag binaries with replacement percentages of 10%, 20%, 30%, 40% and 50% and with a water-to-cement ratio of 0.38. The second series includes ternary mixtures of pumice and silica fume with the same water-to-cement ratio, as shown in Table 2. The name of each design represents the replacement percentage of that material. The cementitious material content in all designs is 500 kg/m^2^. The dry materials were mixed first, and then water and SP were added. The total time of mixing process was about 10 min. After the first three minutes, concrete was rested for about four minutes and then mixed again in the machine for three minutes. After 10 min, the slump flow test was performed.

### 2.3. Non-Destructive Test Method

**Figure 1:**
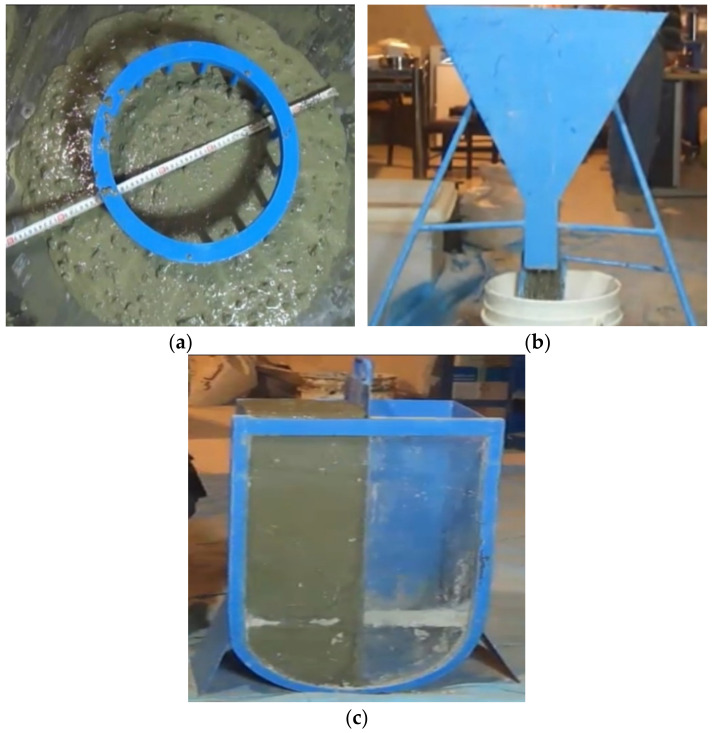
Various test methods: (**a**) J-ring test; (**b**) V-funnel test; (**c**) U-box test.

The slump flow test was performed based on ASTM C1611 to assess the workability of fresh concrete in SCC at different intervals. This test measures the concrete propagation after the funnel removal. Results of slump flow examination indicate the degree of filling ability and SCC stability. To achieve the target slump flow for each mixture, J-ring, V-funnel and U-box tests were performed on fresh SCC according to EFNARC standards. In order to prepare samples for the above tests, at first, each mixture should be stirred for 20 s. Each test methodology is summarized as follows:

(a) As shown in Figure 1a, the J-ring apparatus includes a series of rebars positioned like a cage around the slump cone. The J-ring flow test measures the diameter of flow and the difference between concrete height inside and outside the J-ring (H2–H1). The slump flow test was performed with and without the J-ring in place. The passing ability was measured as an alteration in slump flow.
(b) The flow-ability of concrete with aggregate with a maximum size of 20 mm was measured by the V-funnel flow test, as indicated in Figure 1b. The apparatus includes a funnel with 12 L concrete capacity. The workability of concrete was determined by measuring the time taken by the concrete to flow from the V-funnel after 10 s and 5 min of preparing concrete. Once segregation occurs in concrete, the flow time of concrete increases significantly.
(c) The U-box apparatus (Figure 1c) includes a vessel, which is divided into two compartments by a wall located in the middle. The middle wall includes a sliding gate, which can be lifted. To conduct the U-box test, the left-hand section is filled with about 20 L concrete and then, the gate is lifted to allow the fresh concrete to freely flow into the other section. The result is reported by measuring the concrete heights in the two sections and calculating the difference (H2–H1).

## 3. Test Results

### 3.1. Fresh Concrete Properties

**Figure 2:**
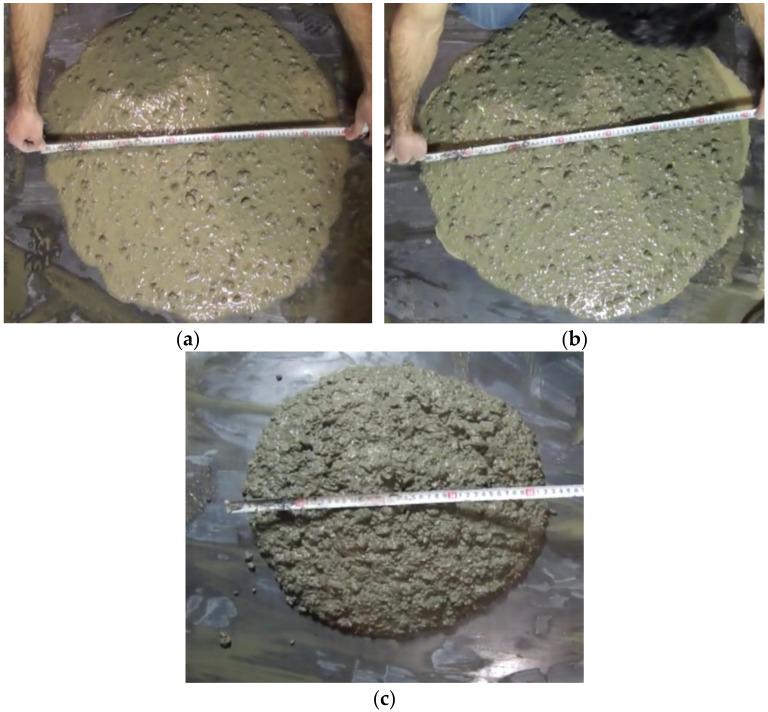
Different slump values in: (**a**) Instant slump, (**b**) 10 min slump, (**c**) 50 min slump (T50).

In binary mixes of slag and pumice, slump flow drop decreases with increasing replacement percentage. Figure 2a,b indicates an increasing trend in slump flow from the initial moment until 10 min after the beginning of the first mixing, but Figure 2c depicts the slump loss at the fiftieth minute. The experiment was repeated more than four times for different percentages to increase the accuracy and confirmation of observations, and the same results were observed. The increasing trend of slump flow could be related to the physical properties of pumice particles. It seems that in the first few minutes, the pumice particles are capable of absorbing mixed water and, after a while, the absorbed water is returned to the mixture. This ability of pumice allows the mixture to maintain the water-to-cement ratio and slump flow in the first minutes. However, after several minutes, the workability deteriorates since the slump loss starts within the mixture.

### 3.2. Superplastisizer Consumption

**Figure 3:**
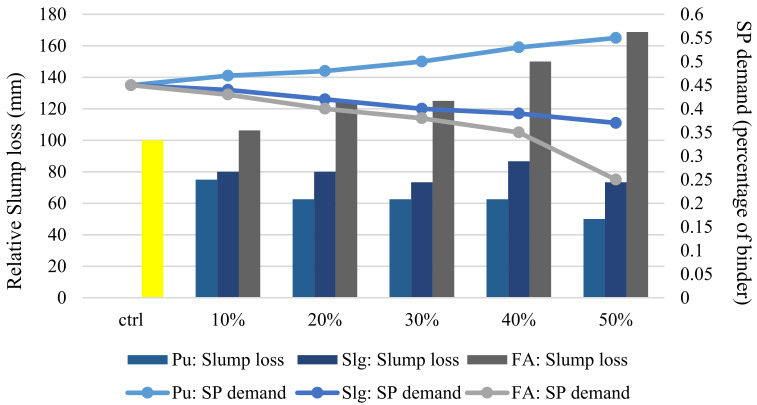
SP consumption and relative slump flow loss in the range 10–50 min for binary designs [19].

**Figure 4:**
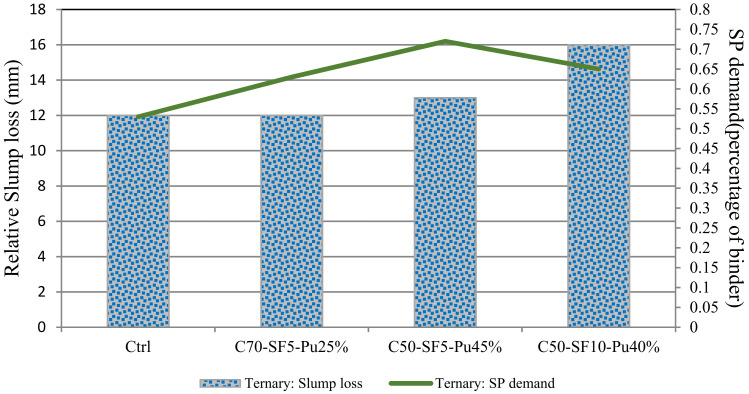
SP consumption and relative slump flow loss in the range 10–50 min for ternary designs [19].

According to [18,19], for better comparison, the SP demand and slump loss between 10 and 50 min for all binary mixes are compared, as can be seen in Figure 3 and Figure 4. All slump losses are converted to relative numbers for improving the analysis. The control specimen of each binary is assumed 100%, and the other designs are proportioned based on each specimen. The amount of SP to achieve a slump of 65 ± 2 is determined in the 10th minute. The results show that more SP is consumed by binary pumice mixes to reach a slump of 65 ± 2, which has a lower slump loss. Increasing the replacement percentage leads to lower consumption of SP.

At the beginning of the mixing process, compared to other specimens, samples with slag represented lower viscosity due to lower water absorption (glass crystalline particles absorb less water compared to pumice and fly ash). These properties of slag make the mixture highly sensitive to SP where adding only a very small amount of SP more than usual will cause concrete segregation. However, Figure 3 and Figure 4 indicate that slag has a better ability to keep the workability for a longer time in the desired range due to the moderate SP consumption and the reasonable slump loss. On the other hand, fly ash has the lowest SP demand and the highest slump loss due to the spherical shape of its particles, which results in decreasing intergranular fraction and improving mixture fluidity. High alumina oxide (45.9%) in fly ash reduces the curing period and therefore slump flow loss occurs faster. The alumina oxide value is much lower for pumice and slag powder, which is confirmed by the obtained results.

According to the results of binary designs, more SP is used by pumice compared to the other two powders for a single slump flow. SP value also increases with increasing replacement but causes less slump flow. On the other hand, adding silica fume to the mixture only causes slump loss over time and does not have a significant effect on the initial slump and amount of initial SP used. Thus, in the ternary mixes of pumice and silica fume, a mixture containing pumice with a higher percentage of replacement requires more SP. However, its slump would decrease, and the mixture containing more silica fume indicates opposite results. As seen in Figure 4, these explanations are in close agreement with the obtained results.

According to the findings of this section, it can be concluded that there is an inverse relationship between the amount of SP demand and the slump flow loss, which shows that lower SP demand causes higher slump loss. The results also suggest that 30% replacement for pumice and slag is very economical and justifiable in binary designs. In ternary designs, 45% pumice replacement with 5% silica fume could be propitious to the design requirement.

Unlike a binary mix of slag and pumice, fly ash shows the opposite result. Increasing fly ash proportion leads to rising slump loss. This behavior could be related to the fine particles of fly ash compared to the size of cement, pumice and slag aggregates, which provide more surface and lead to higher friction between particles. The increase in slump flow continues by adding silica fume to the ternary mix of pumice and silica fume, which is indicated in Figure 3 and Figure 4. Silica fume with a surface area of 2000 kg/m^2^ has the smallest particle size among all powders. Therefore, it is expected that by increasing the replacement value, a further slump flow drop occurs. However, the results in the thirtieth minute show that the presence of pumice with the increasing trend of slump flow is capable of overcoming the slump flow loss induced by silica fume. In all specimens, the slump flow should not be lower than the initial control specimen.

## 4. Artificial Intelligence Method

### 4.1. Adaptive Neuro-Fuzzy Inference System (ANFIS)

**Figure 5:**
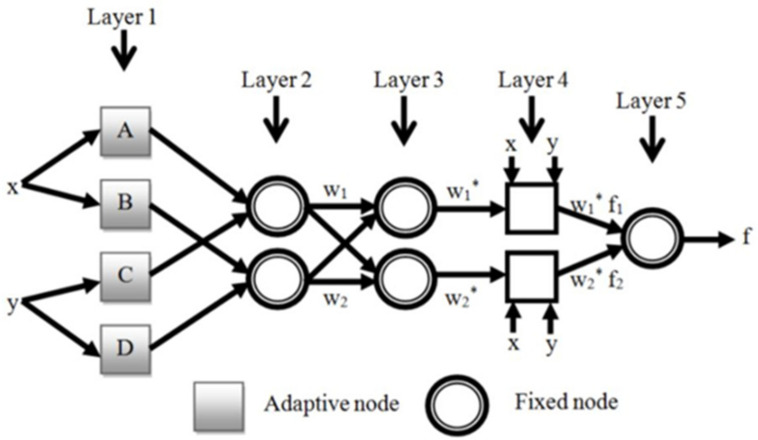
ANFIS basic architecture [43].

ANFIS is a fuzzy Sugeno model placed in an adaptive system framework to build models and then validate developed ones to facilitate training and adaptation. The fuzzy inference system is considered as the core of the ANFIS network. The inputs are received by the first layer and then converted to fuzzy values through the membership function. A combination of least-squares and back-propagation gradient descent methods are used for training parameters of membership function to simulate the given training data set. Figure 5 shows the structure of the whole system formed of five different layers including fuzzy layer, product layer, normalized layer, de-fuzzy layer and total output layer.

### 4.2. Particle Swarm Optimization (PSO)

**Figure 6:**
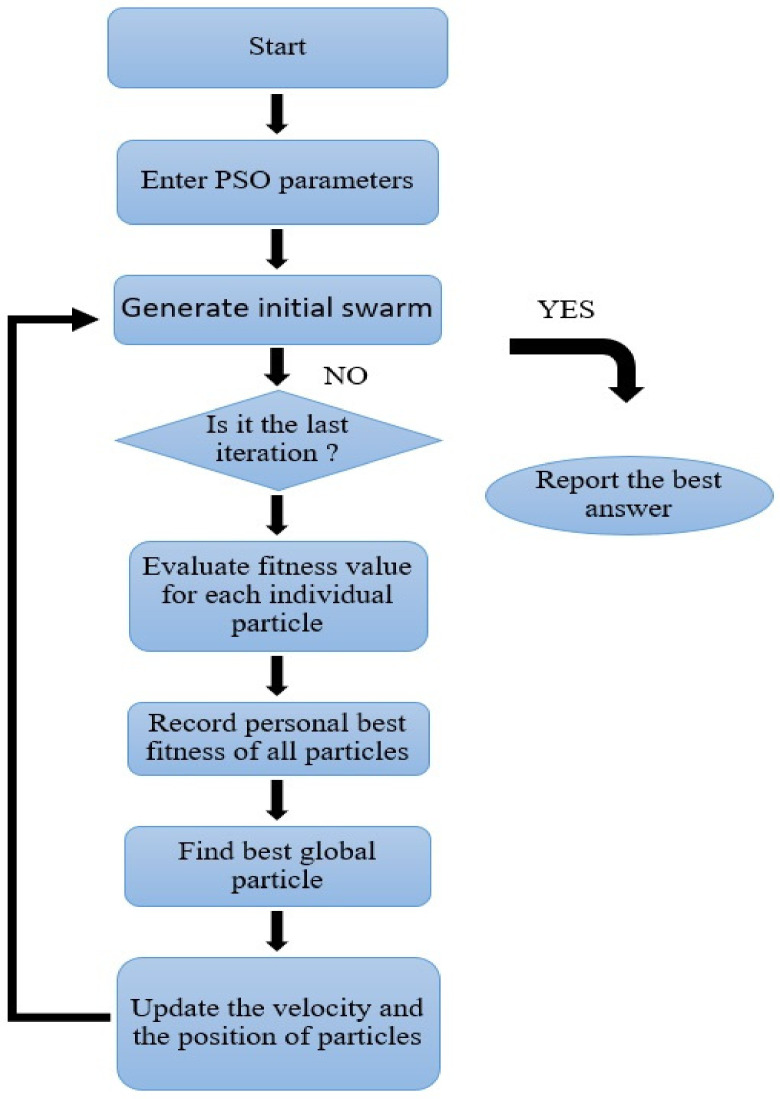
PSO sequential flowchart.

Another component of the swarm intelligence algorithm is particle swarm optimization (PSO) [37,47], inspired by the social behavior of bird flocks or fish schools. This technique is highly comparable to the evolutionary computing methods such as genetic algorithm (GA), which has been indicated in Figure 6. Similar to other population-based intelligence systems, PSO uses a preliminary random solution. The optimal search is achieved by updating the generation without the need for evolutionary operators such as crossovers and mutations. Potential solutions are often referred to as particles in PSO. These particles fly in solution space according to their own experiences and the current best particles.

### 4.3. Ant Colony Optimization (ACO)

**Figure 7:**
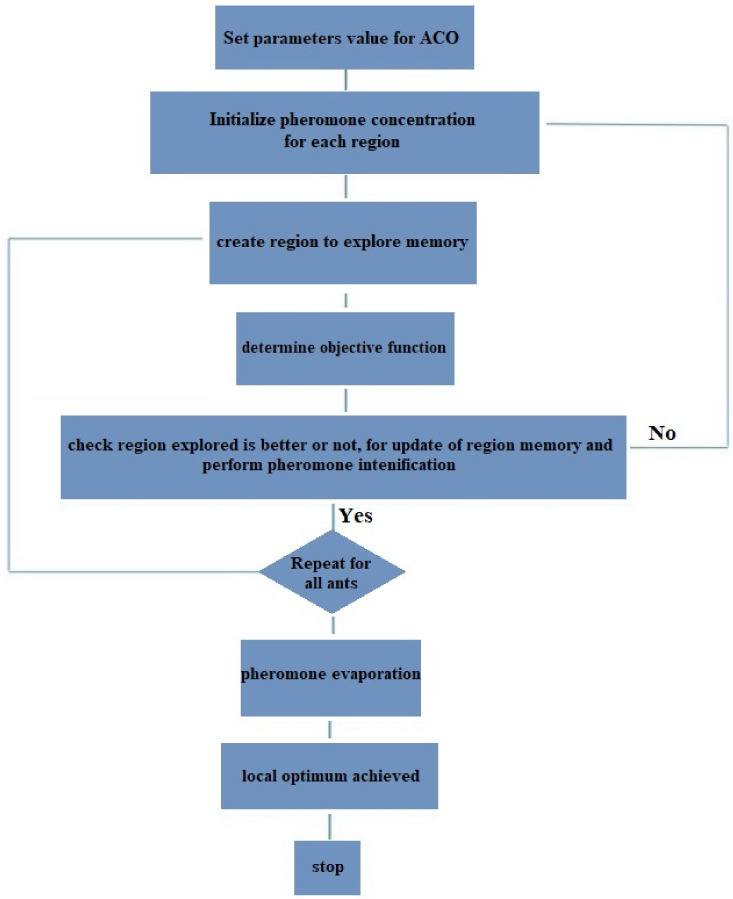
ACO sequential flowchart.

The ant algorithm is a very powerful way to solve hybrid optimization problems. This algorithm is one of the metaheuristic methods inspired by the optimal behavior of ants [48]. Algorithms derived from the ant colony algorithm are a subset of swarm intelligence methods. This methodology is a field of research and study, which investigates concept-inspired algorithms (Swarm Behaviors). Congestion intelligence algorithms consist of a set of simple individual entities that interact and collaborate through self-organizing. Self-organization means the lack of a central control system to control and coordinate the members of a crowded intelligence system. In Figure 7, the employed approach of ACO has been indicated as a sequential flowchart.

### 4.4. Differential Evolution Optimization (DEO)

**Figure 8:**
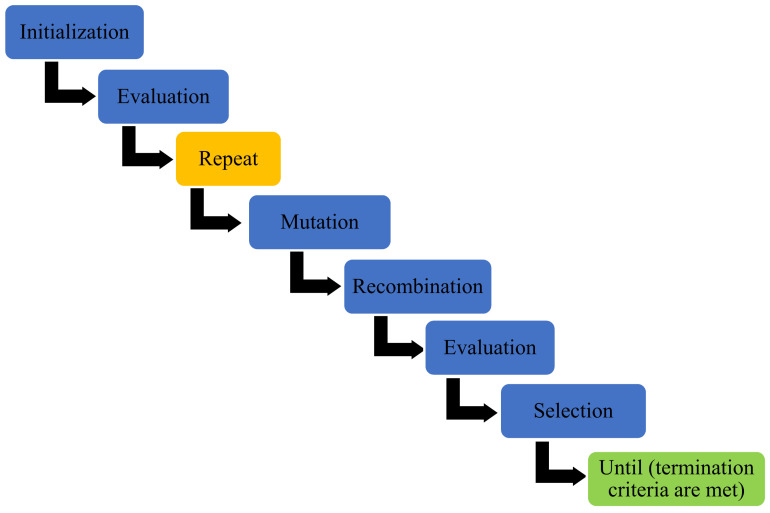
DEO sequential flowchart.

The DEO algorithm proposed by Price and Storn [49] is a population-based algorithm similar to a genetic algorithm (GA) with comparable operators: crossover, mutation and selection. The primary difference is that GAs depend on crossover while DEO is based on mutation operation, which relies on the difference between randomly sampled pairs of solutions in the population. Figure 8 shows the main steps of the DEO algorithm.

### 4.5. Architecture of ANFIS-PSO/ACO/DEO

**Figure 9:**
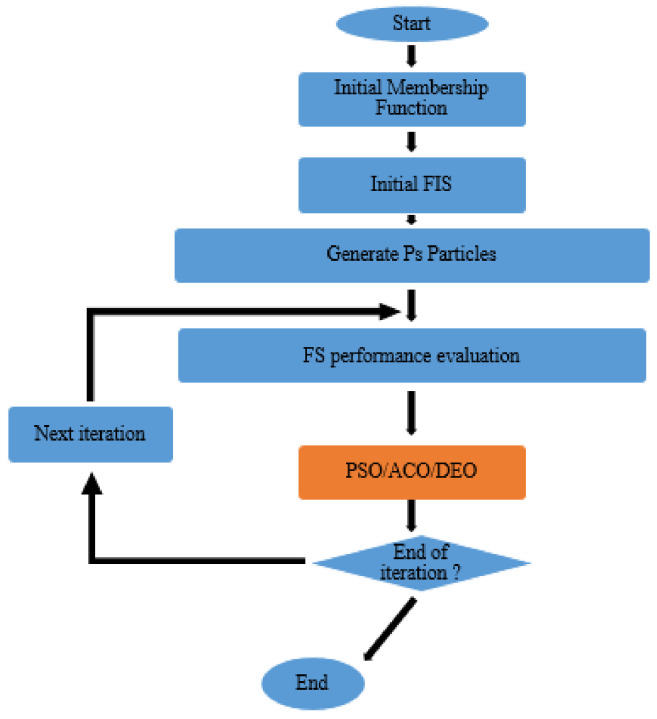
Diagram of the sequential combination of PSO/ACO/DEO and ANFIS.

Figure 9 illustrates the combination of sequential PSO/ACO/DEO and ANFIS [43]. In PSO, a swarm begins with a set of random solutions, and si⇀ represents the position of the particle. Similarly, the particle swarm moves in the problem space, where vi⇀ indicates the velocity of the particle. At every time interval, the function (f) is determined by inputting si⇀ through a hybrid algorithm. Each particle records the best position associated with the best fit obtained at this time in the pi⇀ vector. Pig⇀ tracks the most applicable location for any neighbour member ID. In the generic version of PSO, Pig⇀ represents the most suitable point in the entire population. Depending on the optimal position of the individual pi(t)⇀ and p⇀ig(t) neighborhood, every particle (i) achieves a new velocity, which can be presented by:

v_i⇀(t + 1) = wv_i⇀(t) + c_1 ∅_1⇀·(p_i⇀(t) − x_i⇀(t)) + c_2 ∅_2⇀·(p_i⇀(t) − x_i⇀(t))(1)

where w represents the inertia weight, c1 and c2 are the positive acceleration coefficients, ∅1⇀ and ∅2⇀ denote uniformly distributed random vectors in [0,1], where each dimension tries a random value. The vi⇀ limit in the [−vmax⇀,vmax⇀] series depends on the problem. If the velocity exceeds the above limit, the velocity may be rescheduled within the appropriate limits. Based on their velocity, each particle changes its position according to the following equation:

s_i⇀(t + 1) = s_i⇀(t) + v_i⇀(t + 1)(2)

Based on vi⇀ and si⇀, the particle population tends to cluster around the best.

### 4.6. Performance Evaluation

The data obtained by Equation (2) requires to be normalized, since the problem of prediction is non-linear. Hence, pre-processing and post-processing can be carried out [50], and the input data is normalized in the interval of −1 and 1 through the following formulas:

(3) $$x_{i}=\frac{x_{io}-x_{min}}{x_{max}-x_{min}}\times 2-1$$

(4) $$y_{i}=\frac{y_{io}-y_{min}}{y_{max}-y_{min}}\times 2-1$$

where $x_{io}$ and $x_{i}$ are the *i*-th component of each input vector before and after normalization, respectively, and $y_{io}$ and $y_{i}$ are the i^th component of the output vector before and after normalization, respectively. Also, $x_{min}$, $x_{max}$, $y_{min}$, and $y_{max}$ are the minimum and maximum values of each input and output vector, respectively.

By evaluating the performance of the models, the training phase is composed of 70% of the data, and the other 30% is assigned to the testing phase. Then, root mean square error (*RMSE*), Pearson correlation coefficient (*r*) and determination coefficient (R^2^) are used as performance indices of the models. These indicators are presented as follows:

(5) $$RMSE=\sqrt{\frac{\sum_{k=1}^{S}{\left(P_{k}-T_{k}\right)}^{2}}{S}}$$

(6) $$r=\frac{S\left(\sum_{k=1}^{S}T_{k}\times P_{k}\right)-\left(\sum_{k=1}^{S}T_{k}\right)\times \left(\sum_{k=1}^{S}P_{k}\right)}{\sqrt{\left(S\sum_{k=1}^{S}T_{k}^{2}-{\left(\sum_{k=1}^{S}T_{k}\right)}^{2}\right)\times \left(S\sum_{k=1}^{S}{P_{k}}^{2}-{\left(\sum_{k=1}^{S}P_{k}\right)}^{2}\right)}}$$

(7) $$R^{2}=\frac{{\left[\sum_{k=1}^{S}\left(T_{k}-\bar{T_{k}}\right)\cdot \left(P_{k}-\bar{P_{k}}\right)\right]}^{2}}{\sum_{k=1}^{S}\left(T_{k}-\bar{T_{k}}\right)\cdot \sum_{k=1}^{S}\left(P_{k}-\bar{P_{k}}\right)}$$

where *P* and *T* are the predicted and target values, respectively, and *S* is the total number of training or testing samples. The above equations were written in MATLAB environment so that at the first stage, predicted and target values were placed in an individual file. Secondly, equations were filled with corresponding values by written the codes and finally, correlation coefficient values were derived from the MATLAB software and placed in another file.

### 4.7. Statistical Data

The obtained results from experiments on concrete specimens include 26 data sets. The results of the J-ring, U-box, V-funnel tests, slump value in the third minute and slump value in the fiftieth minute were the inputs of the models, and the SP demand was considered as the output. Table 3 shows some details of the dataset, and Table 4 represents the statistical data used for hybrid models.

### 4.8. Model Development

Table 5 summarizes the information for the 26-row data set obtained from experiments, which are used in the current research.

ANFIS-PSO, ANFIS-ACO and ANFIS-DEO as hybrid algorithms were used for the first time. Each algorithm has parameters that can be changed to achieve the desired results. The parameters for PSO algorithm are population size, iterations, inertia weight, damping ratio, personal and global learning coefficient. The ACO algorithm parameters are population size, iterations, sample size, intensification factor and deviation-distance ratio. The DEO algorithm parameters are size, iterations, lower bound of scaling factor, upper bound of scaling factor and crossover probability. These parameters should also be determined in hybrid algorithms. The hybrid models were several times run for different parameters and tuned to achieve the best performance. Table 6, Table 7 and Table 8 provide the optimal settings for each algorithm that resulted from many experiments.

## 5. Discussion of Results

**Figure 10:**
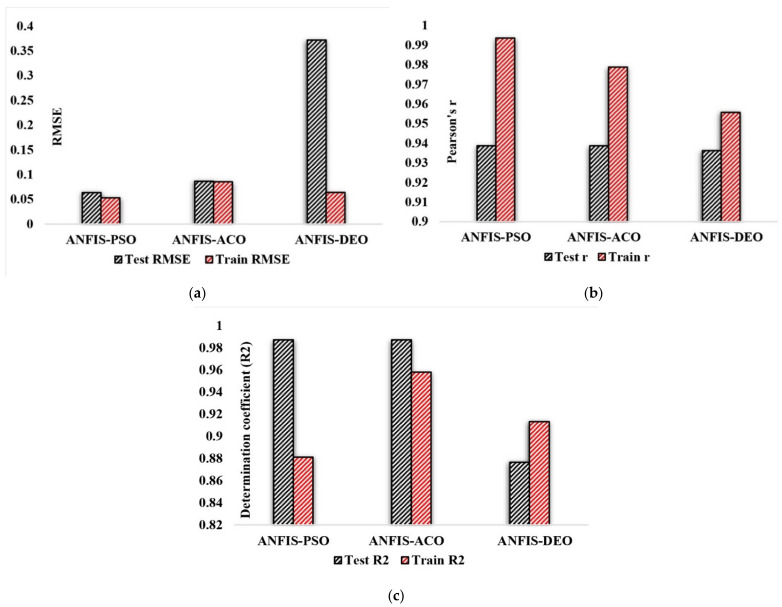
Comparison of evaluation parameters: (**a**) RMSE, (**b**) Pearson correlation coefficient, (**c**) determination coefficient.

ANFIS was integrated with three metaheuristic algorithms, including PSO, ACO and DEO, to calculate the amount of required superplasticizer in concrete. The ANFIS parameters were constant in all three states. Cluster account has chosen 10% of data for training (70% of the input data) and testing (30% of the input data). The results show that all three hybrid neural networks are reliable. However, Figure 10 and Table 9 show that the ANFIS-DEO algorithm has slightly overtrained. After performing several experiments that combined different input states as well as various states of neural network algorithm parameters, the best results were selected for each algorithm as follows:

As shown in Table 9, the performance parameters in the testing phase for ANFIS-PSO model are RMSE = 0.0633, r = 0.9387 and R^2^ = 0.9871, for ANFIS-ACO are RMSE = 0.0864, r = 0.9073 and R^2^ = 0.8231, and for ANFIS-DEO are RMSE = 0.3717, r = 0.9362 and R^2^ = 0.8765. In the training phase, these parameters for ANFIS-PSO were RMSE = 0.0529, r = 0.9935 and R^2^ = 0.8811 while ANFIS-ACO provided RMSE = 0.0854, r = 0.9787 and R^2^ = 0.9579, and ANFIS-DEO obtained RMSE = 0.0638, r = 0.9556 and R^2^ = 0.9132.

Given that the best result for RMSE is the lowest one, and for r, the best positive correlation coefficient is 1.0, the numbers closer to 1.0 are; therefore, better results. Considering all the conditions stated above, it is clear that the ANFIS-PSO algorithm performs better than the other two algorithms in the testing phase, as shown in Figure 10.

**Figure 11:**
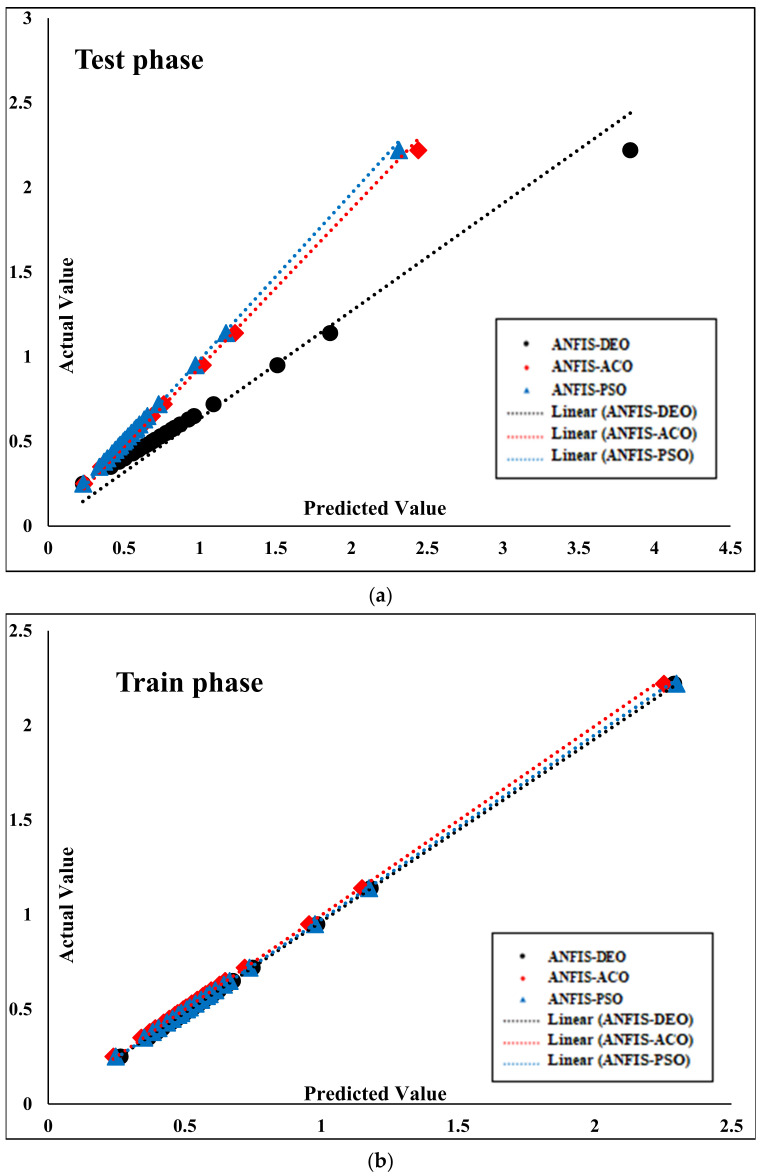
Comparison of regression lines: (**a**) Testing phase, (**b**) training phase.

Figure 11 shows comparable slope lines of both training and testing phases of employed algorithms. According to Figure 11a, ANFIS-PSO and ANFIS-ACO algorithms produced very good results and performed better in the training phase. By observing the slope of the lines in Figure 11b, it is clear that ANFIS-PSO and ANFIS-ACO algorithms are able to find a better relationship between the input and output data and also provide appropriate results in the testing phase.

**Figure 12:**
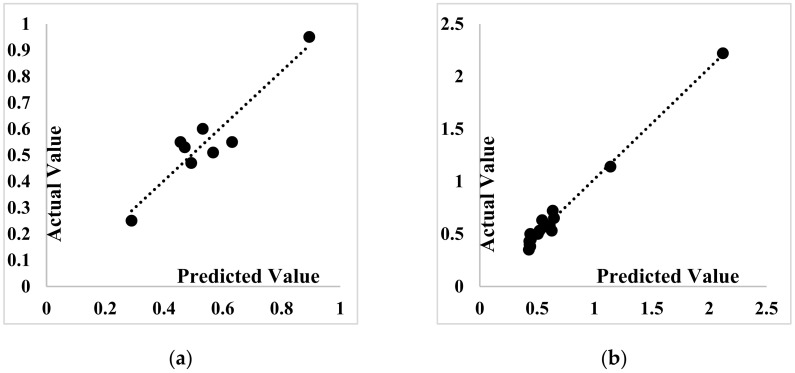
Scatter plot of the SP demand prediction for ANFIS-PSO: (**a**) Testing phase (**b**) Training phase.

**Figure 13:**
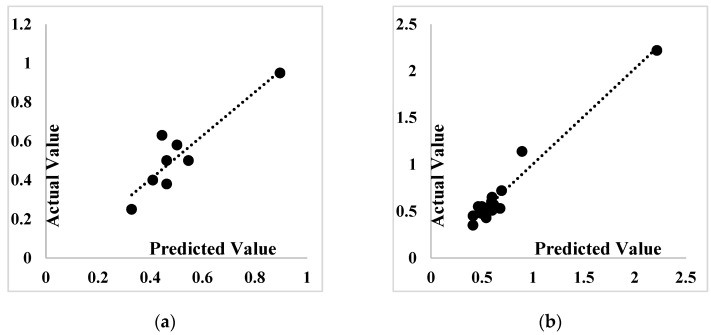
Scatter plot of the SP demand prediction for ANFIS-ACO: (**a**) Testing phase, (**b**) training phase.

**Figure 14:**
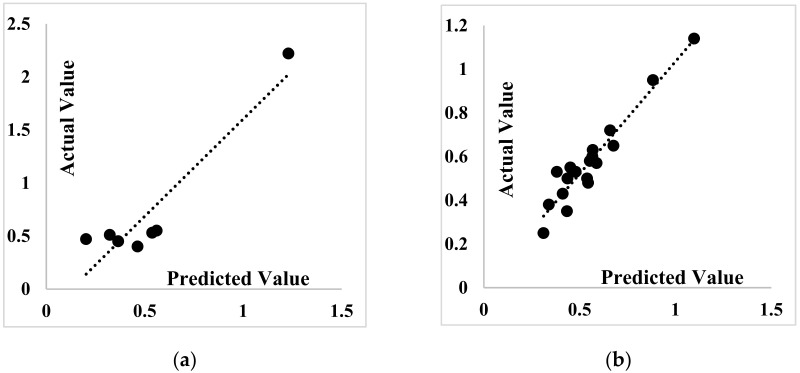
Scatter plot of the SP demand prediction for ANFIS-DEO: (**a**) Testing phase, (**b**) training phase.

The calculated regression equations for each algorithm are shown in Table 10, and the corresponding graphs are indicated in Figure 12, Figure 13 and Figure 14. In both training and testing phases, the ANFIS-PSO algorithm performs better than ANFIS-ACO and ANFIS-DEO.

**Figure 15:**
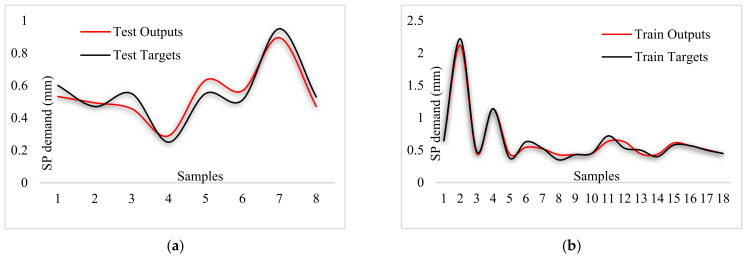
Comparison of outputs and targets for ANFIS-PSO: (**a**) Testing phase, (**b**) training phase.

**Figure 16:**
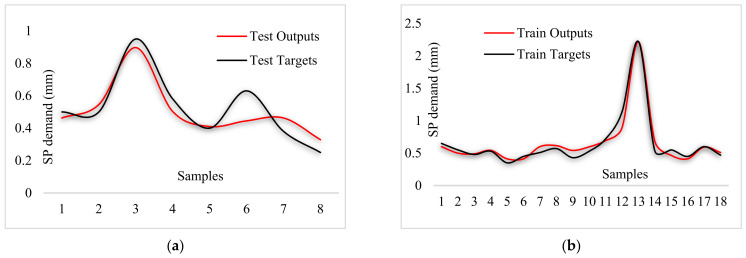
Comparison of outputs and targets for ANFIS-ACO: (**a**) Testing phase, (**b**) training phase.

**Figure 17:**
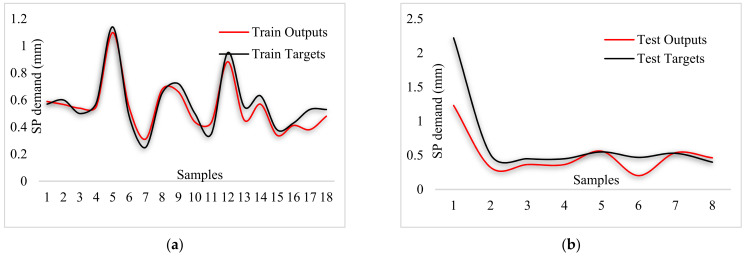
Comparison of outputs and targets for ANFIS-DEO: (**a**) Testing phase, (**b**) training phase.

By comparing Figure 15, Figure 16 and Figure 17, the better capability of the ANFIS-PSO model for predicting each of the measured values of the test samples can be observed, which is more accurate than other models.

According to Table 11, error numbers indicate the accuracy of the results in which the smaller value implies the accurate process. The standard deviation value directly illustrates the behavior of the results in accordance with the mean value, where the smaller the standard deviation number, the closer the results are to the mean value. Therefore, the ANFIS-PSO algorithm performs better in both the testing and training phases.

**Figure 18:**
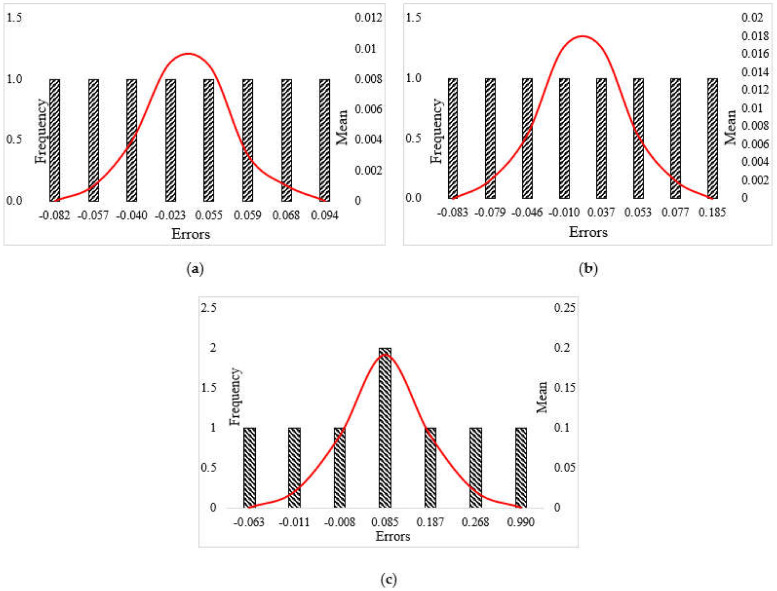
Error histograms for: (**a**) ANFIS-PSO, (**b**) ANFIS-ACO and (**c**) ANFIS-DEO algorithms.

Figure 18 shows the error diagrams with corresponding envelop for all hybrid algorithms. It can be seen that the ANFIS-PSO has a smaller error interval, indicating the least error mean of this model.

As discussed comprehensively, ANFIS can be considered as a reliable predictive method to be integrated with other metaheuristics algorithms, like previous studies [28,43,44] which have used hybrid algorithms to solve non-linear relationships between input and output variables.

## 6. Conclusions

Self-consolidating concrete (SCC) requires a higher dosage of cement compared to normal concrete, which is a controversial issue from an environmental point of view. In order to solve this problem, researchers studied different natural and synthetic powders as partial replacements. The remaining workability of SCC is an important factor to achieve a sustainable mix design, which is directly related to superplasticizer (SP) content in the SCC mix. SP demand is an optimum value of a dosage, which is derived from non-destructive tests. This study was aimed to investigate the application of artificial intelligence algorithms to overcome the difficulties in the prediction of SP demand through non-destructive tests including J-ring, U-box, V-funnel, 3 min slump and T50. To this end, ANFIS was integrated with three metaheuristic algorithms, namely PSO, ACO and DEO. The most important results can be summarized as follows:

- The developed hybrid algorithms were trained by the collected dataset and, finally, the SP demand values were predicted for specified SCC mixes. In terms of performance parameters, all hybrid algorithms obtained promising results.
- Compared to other proposed algorithms, ANFIS-PSO represented the best evaluation criteria including RMSE = 0.0633, r = 0.9387 and R^2^ = 0.9871 in the testing phase and RMSE = 0.0529, r = 0.9935 and R^2^ = 0.8811 in the training phase. Prediction errors were also in an acceptable range where the ANFIS-PSO indicated the lowest ones. Additionally, test and train results of all three algorithms were presented in an analogous regression diagram for a better understanding of the accuracy and eligibility of each technique.
- It was found that metaheuristic algorithms, especially the PSO technique, are able to cover the prediction problems of non-linear data. In general, the best performance of hybrid models was obtained for ANFIS-PSO, ANFIS-ACO and ANFIS-DEO, respectively. In addition, it seems that ANFIS can be a good base for other metaheuristic optimization algorithms such as genetic algorithm, firefly and bee colony.

## Figures and Tables

**Table 1:** The chemical components of Portland cement and other cementitious materials.

Components (%)	Cement	FA ^1^	Pumice	Slag	SF ^1^
SiO_2_	22.42	62.8	44.13	33.1	86.2
Al_2_O_3_	4.68	45.9	16.71	13.8	1.44
Fe_2_O_3_	3.68	0.92	1.72	3.12	0.2
CaO	63.25	2.60	11.09	40.7	3.06
MgO	3.63	1.40	1.95	8.70	1.32
SO_3_	1.74	0.49	0.39	0.60	0.34
Specific gravity (kg/m^3^)	3160	2200	2850	2850	2350
Blaine (m^2^/kg)	290	260	320	445	20,000

^1^ FA: Fly ash, SF: Silica fume.

**Table 2:** Mix proportion of self-consolidating concrete.

Cement Material	Powder (kg/m^3^)					Aggregate (kg/m^3^)		
	Pumice	Silica Fume	Slag	Fly Ash	OPC	Gravel	Sand	Water (kg/m^3^)
Control	–	–	–	–	500	1070	580	191
FA10	–	–	–	50	450	1063	590	191
FA20	–	–	–	100	400	1052	584	191
FA30	–	–	–	150	350	1040	578	191
FA40	–	–	–	200	300	1029	571	191
FA50	–	–	–	250	250	1017	565	191
Pu10	50	–	–	–	450	1072	595	191
Pu20	100	–	–	–	400	1069	594	191
Pu30	150	–	–	–	350	1066	592	191
Pu40	200	–	–	–	300	1063	590	191
Pu50	250	–	–	–	250	1060	589	191
Slg10	–	–	50	–	450	1072	595	191
Slg20	–	–	100	–	400	1069	594	191
Slg30	–	–	150	–	350	1066	592	191
Slg40	–	–	200	–	300	1063	590	191
Slg50	–	–	250	–	250	1069	580	191
Pu25-SF5	125	25	–	–	350	1063	590	191
Pu45-SF5	225	25	–	–	250	1057	587	191
Pu40-SF10	250	50	–	–	200	1051	584	191

**Table 3:** Details of the input and output variables.

Inputs and Outputs	Variables	Minimum	Maximum	Mean Value	Standard Deviation
Input 1	J-ring (mm)	0.70	6.15	2.71	1.44
Input 2	U-box (mm)	0.50	25.00	4.12	5.19
Input 3	V-funnel (s)	5.00	60.00	8.73	10.58
Input 4	3 min Slump (mm)	41.00	66.00	52.85	6.72
Input 5	50 min Slump (mm)	43.00	62.00	55.81	4.49
Output	SP demand (mg)	0.25	2.22	0.61	0.37

**Table 4:** Statistical data.

Input 1	Input 2	Input 3	Input 4	Input 5	Output
J-Ring (mm)	U-Box (mm)	V-Funnel (s)	3 min Slump (mm)	50 min Slump (mm)	SP Demand (mg)
1	0.5	5	52	58	0.45
1	1	5	66	59	0.47
2	1	5	46	58	0.48
2	1.5	7	55	62	0.5
3	2	8	52	60	0.53
3	2.5	9	55	60	0.55
1	0.5	5	52	58	0.45
1	1	5	66	59	0.43
1	1	5	46	58	0.4
2	1.5	7	55	62	0.38
3	2	7	52	60	0.35
3	2	8	55	55	0.25
0.7	3	6	42	50	0.58
5	12	9	55	55	0.57
2.25	8	8	55	55	0.55
2.5	4	10	58	56	0.53
2	3	7	66	50	0.51
3.15	5.5	7	58	56	0.50
4.25	4	5	53	52	0.53
3.05	10	5	46	52	0.63
3.1	1	6	53	52	0.72
3.28	7	5	44	49	0.65
2.75	2	6	53	59	0.60
3.65	2	7	41	57	0.95
5.75	4	10	52	56	1.14
6.15	25	60	46	43	2.22

**Table 5:** Inputs and outputs at a glance.

Inputs and Outputs	Minimum	Maximum	Average
J-ring (mm)	0.70	6.15	2.71
U-box (mm)	0.50	25.00	4.12
V-funnel (s)	5.00	60.00	8.73
Slump 3 min (mm)	41.00	66.00	52.85
Slump 50 min (mm)	43.00	62.00	55.80
SP demand (mg)	0.25	2.22	0.61

**Table 6:** Parameter characteristics used for PSO.

FIS Clusters	Population Size	Iterations	Inertia Weight	Damping Ratio	Learning Coefficient	
					Personal	Global
10	240	150	1	0.99	1	2

**Table 7:** Parameter characteristics used for ACO.

FIS Clusters	Population Size	Iterations	Sample Size	Intensification Factor	Deviation-Distance Ratio
10	240	150	480	0.5	1

**Table 8:** Parameter characteristics used for DEO.

FIS Clusters	Population Size	Iterations	Lower Bound of Scaling Factor	Upper Bound of Scaling Factor	Crossover Probability
10	240	150	0.2	0.8	0.1

**Table 9:** The calculated accuracy criteria for the performance of the implemented models.

Ensemble Model	Network Result					
	Training Phase			Testing Phase		
	RMSE	r	R^2^	RMSE	r	R^2^
ANFIS-PSO	0.0529	0.9935	0.8811	0.0633	0.9387	0.9871
ANFIS-ACO	0.0854	0.9787	0.9579	0.0864	0.9073	0.8231
ANFIS-DEO	0.0638	0.9556	0.9132	0.3717	0.9362	0.8765

**Table 10:** The calculated regression equation of the implemented models.

Ensemble Model	Network Result	
	Training Phase	Testing Phase
	Regression Equation	Regression Equation
ANFIS-PSO	y = 1.0414x − 0.0133	y = 1.0571x − 0.033
ANFIS-ACO	y = 1.0224x − 0.0174	y = 1.1189x − 0.0434
ANFIS-DEO	y = 1.0282x + 0.0073	y = 1.8328x − 0.2297

**Table 11:** The calculated errors of the implemented models.

Ensemble Model	Network Result			
	Training Phase		Testing Phase	
	Errors Mean	Standard Deviation	Errors Mean	Standard Deviation
ANFIS-PSO	0.0033	0.0543	0.0092	0.0669
ANFIS-ACO	−0.0027	0.0878	0.0169	0.0905
ANFIS-DEO	0.0228	0.0613	0.1916	0.3405

## Data Availability

Data sharing is not applicable to this article.
